# Deep learning radiomics based prediction of axillary lymph node metastasis in breast cancer

**DOI:** 10.1038/s41523-024-00628-4

**Published:** 2024-03-12

**Authors:** Han Liu, Liwen Zou, Nan Xu, Haiyun Shen, Yu Zhang, Peng Wan, Baojie Wen, Xiaojing Zhang, Yuhong He, Luying Gui, Wentao Kong

**Affiliations:** 1 Department of Ultrasound, Nanjing Drum Tower Hospital, Affiliated Hospital of Medical School, Nanjing University, Nanjing, 210002 China; 2 Department of Mathematics, Nanjing University, Nanjing, 210008 China; 3 Department of Ultrasound, Jinling Hospital, Medical School of Nanjing University/General Hospital of Eastern Theater Command, Nanjing, 210002 China; 4 College of Computer Science and Technology, Nanjing University of Aeronautics and Astronautics, MIIT Key Laboratory of Pattern Analysis and Machine Intelligence, Nanjing, 211106 China; 5 Department of Ultrasound, Taizhou Hospital Affiliated to Nanjing University of Chinese Medicine, Taizhou, 225300 China; 6 School of Mathematics and Statistics, Nanjing University of Science and Technology, Nanjing, 210094 China

**Keywords:** Breast cancer, Medical research

## Abstract

This study aimed to develop and validate a deep learning radiomics nomogram (DLRN) for the preoperative evaluation of axillary lymph node (ALN) metastasis status in patients with a newly diagnosed unifocal breast cancer. A total of 883 eligible patients with breast cancer who underwent preoperative breast and axillary ultrasound were retrospectively enrolled between April 1, 2016, and June 30, 2022. The training cohort comprised 621 patients from Hospital I; the external validation cohorts comprised 112, 87, and 63 patients from Hospitals II, III, and IV, respectively. A DLR signature was created based on the deep learning and handcrafted features, and the DLRN was then developed based on the signature and four independent clinical parameters. The DLRN exhibited good performance, yielding areas under the receiver operating characteristic curve (AUC) of 0.914, 0.929, and 0.952 in the three external validation cohorts, respectively. Decision curve and calibration curve analyses demonstrated the favorable clinical value and calibration of the nomogram. In addition, the DLRN outperformed five experienced radiologists in all cohorts. This has the potential to guide appropriate management of the axilla in patients with breast cancer, including avoiding overtreatment.

## Introduction

Breast cancer ranks as the second leading cause of cancer-related mortality in women and has a high metastasis rate of 20–30%^1^. As the most frequent site of metastasis, the status of axillary lymph nodes (ALNs) is pivotal for pathological staging, prognosis, and treatment guidance including determining neoadjuvant or adjuvant therapy and surgical planning for patients^2^. Currently, ALN dissection and sentinel lymph node (SLN) biopsy are the standard methods for determining the metastatic status of ALNs^3–5^. Nevertheless, both methods are invasive and may lead to postoperative morbidity, such as arm numbness and upper limb edema^6,7^. In addition, 70–80% of patients who undergo SLN biopsy exhibit negative SLNs, indicating the high probability of overtreatment with unnecessary SLN biopsy^8,9^. Therefore, an accurate, non-invasive approach to predict ALN metastasis would be part of optimal treatment planning in patients with newly diagnosed breast cancer.

Ultrasound represents a widely used imaging tool for preoperative assessment of ALNs in patients with breast cancer. On axillary ultrasound, ALNs with features such as longest/shortest axis ratio < 2, cortical thickening, and loss of fatty hilum in the node are suspected to be malignant^10^. Previous studies suggested that integration of suspicious features on axillary ultrasound and other clinical factors had the potential to predict ALN metastasis^11^. However, the performance is unsatisfactory, with a limited area under the receiver operating characteristic (ROC) curve (AUC) of 0.585–0.719^12^. Additionally, ALNs with suspicious morphology often undergo ultrasound-guided needle biopsy to assist in preoperative planning. However, nearly 35% of metastatic ALNs do not show any suspicious features^13^, introducing a limitation in the assessment of ALN status using axillary ultrasound.

Several studies have developed noninvasive methods to determine the metastatic status of ALNs, including several clinical nomograms^14–16^. However, few of these studies have taken into account information derived from preoperative imaging of the lesion, and some have incorporated factors that can only be obtained postoperatively. Recently, radiomics has been an effective technology in clinic by converting medical image data into high-throughput imaging features^17^. The latest studies have revealed improvements in assessing ALN status using conventional radiomics (CR) analysis of primary tumors with mammography, ultrasound, and magnetic resonance imaging^18–20^. However, the limitations of handcrafted features in CR lie in manual labeling and their inability to conform to a specific task^21^. In contrast, deep learning radiomics (DLR)^22^ is an innovative method that can learn end-to-end and automatically discover multiple levels of representations for the specific prediction tasks. The DLR process includes feeding raw machine data, such as medical images, and allowing them to learn feature representations, quantify information from images, and discover vectors for prediction tasks using multiple layers of features^23^. With technological advancements, the application of DLR in breast cancer imaging has rapidly increased, including prediction of ALN status^2,24–26^. However, the clinical utility of these studies remains uncertain as they contained limited sample sizes, lacked external validation, and neglected important clinical information^27^, which are crucial for classification accuracy^28^. Therefore, we planned to integrate DLR features extracted from breast ultrasound and preoperative clinical parameters to improve the model performance with a large sample size.

In this study, we developed a deep learning radiomics nomogram (DLRN) based on breast ultrasound, which is a convenient, radiation-free, and favorably repeatable examination for breast cancer^29,30^, to access ALN status preoperatively. The predictive performance of the DLRN was validated using three external validation cohorts (EVCs). Ultimately, the results indicated that DLRN could detect the metastatic risk of ALNs better than the clinical model and radiologists, enable individualized surgical approaches for the axilla, and minimize overtreatment.

## Results

### Baseline characteristics

Table 1 summarizes the clinical parameters of the 883 patients with breast cancer in the four hospitals. According to the results of SLN biopsy or ALN dissection, the ALN metastatic rates were 34.6%, 40.2%, 40.2%, and 50.8% in TC, EVC1, EVC2, and EVC3, respectively. The median BMI were 26.3, 25.7, 25.2, and 25.7 in the four cohorts, respectively. The Kappa values of the BI-RADS category were 0.957 and 1 for inter- and intra-observer agreements, respectively (both *P* < 0.001). For US-ALN, the Kappa values were 0.936 and 1, respectively (both *P* < 0.001). The metastatic status of ALN showed a significant difference between the TC and EVC3 (34.6% vs. 50.8%, *P* < 0.001). Other detailed differences in specific clinical parameters are described in Supplementary Notes.

**Table 1:** Participant baseline characteristics in four cohorts

Characteristic	TC (*n* = 621)	EVC1 (*n* = 112)	EVC2 (*n* = 87)	EVC3 (*n* = 63)
Age (years)	54.50 ± 11.51	55.19 ± 10.65	53.93 ± 11.94	54.78 ± 9.83
Ultrasound size (cm)	2.24 ± 1.06	2.26 ± 1.12	2.17 ± 1.21	2.22 ± 0.98
BMI				
<25	237 (38.16%)	36 (32.14%)	26 (29.89%)	21 (33.33%)
25–30	172 (27.70%)	36 (32.14%)	27 (31.03%)	22 (34.92%)
>30	174 (28.02%)	35 (31.25%)	32 (36.78%)	14 (22.22%)
Not appliable	38 (6.12%)	5 (4.46%)	2 (2.30%)	6 (9.52%)
BI-RADS category				
4A	172 (27.70%)	18 (16.07%)	6 (6.90%)	13 (20.63%)
4B	144 (23.19%)	35 (31.25%)	12 (13.79%)	21 (33.33%)
4C	133 (21.42%)	8 (7.14%)	12 (13.79%)	18 (28.57%)
5	172 (27.70%)	51 (45.54%)	57 (65.52%)	11 (17.46%)
Tumor location				
UOQ	130 (20.93%)	21 (18.75%)	25 (28.74%)	31 (49.21%)
LOQ	61 (9.82%)	12 (10.71%)	6 (6.90%)	12 (19.05%)
UIQ	163 (26.25%)	38 (33.93%)	25 (28.74%)	6 (9.52%)
LIQ	66 (10.63%)	26 (23.21%)	13 (14.94%)	13 (20.63%)
Central	201 (32.37%)	15 (13.39%)	18 (20.69%)	1 (1.59%)
Nuclear grade				
I	96 (15.46%)	5 (4.46%)	13 (14.94%)	10 (15.87%)
II	298 (47.99%)	67 (59.82%)	49 (56.32%)	37 (58.73%)
III	227 (36.55%)	40 (35.71%)	25 (28.74%)	16 (25.40%)
Tumor classification				
Noninvasive carcinoma	59 (9.51%)	4 (3.57%)	7 (8.05%)	2 (3.17%)
Invasive carcinoma				
NST	489 (78.74%)	97 (86.61%)	71 (81.61%)	60 (95.24%)
ST	43 (6.92%)	8 (7.14%)	6 (6.90%)	0 (0.00%)
Rare carcinoma	30 (4.83%)	3 (2.68%)	3 (3.45%)	1 (1.59%)
ER				
Positive	451 (72.62%)	83 (74.11%)	64 (73.56%)	35 (55.56%)
Negative	170 (27.38%)	29 (25.89%)	23 (26.44%)	28 (44.44%)
PR				
Positive	402 (64.73%)	78 (69.64%)	52 (59.77%)	42 (66.67%)
Negative	219 (35.27%)	34 (30.36%)	35 (40.23%)	21 (33.33%)
HER2				
Positive	520 (83.73%)	34 (30.36%)	66 (75.86%)	48 (76.19%)
Negative	101 (16.26%)	78 (69.64%)	21 (24.14%)	15 (23.81%)
Ki-67				
Positive	514 (82.77%)	90 (80.36%)	66 (75.86%)	47 (74.60%)
Negative	107 (17.23%)	22 (19.64%)	21 (24.14%)	16 (25.40%)
Surrogate subtype				
Luminal A-like	248 (39.94%)	16 (14.29%)	6 (6.90%)	1 (1.59%)
Luminal B-like	216 (34.78%)	71 (63.39%)	58 (66.67%)	39 (61.90%)
HER2-overexpression	127 (20.45%)	11 (9.82%)	20 (22.99%)	15 (23.81%)
Triple negative	30 (4.83%)	14 (12.50%)	3 (3.45%)	8 (12.70%)
US-ALN				
Suspicious	242 (38.97%)	37 (33.04%)	44 (50.57%)	22 (34.92%)
Non-suspicious	379 (61.03%)	75 (66.96%)	43 (49.43%)	41 (65.08%)
ALN metastasis				
Positive	215 (34.62%)	45 (40.18%)	35 (40.23%)	32 (50.79%)
Negative	406 (65.38%)	67 (59.82%)	52 (59.77%)	31 (49.21%)

*TC* training cohort, *EVC* external validation cohort, *BMI* body mass index, *UOQ* upper outer quadrant, *LOQ* lower outer quadrant, *UIQ* upper inner quadrant, *LIQ* lower inner quadrant, *NST* no special type, *ST* special type, *ER* estrogen receptor, *PR* progesterone receptor, *HER2* human epidermal growth factor receptor-2, *US-ALN* axillary lymph nodes status reported by axillary ultrasound.

### DLR signature construction and validation

In total, 544 handcrafted and 2048 deep learning features were extracted for each patient. Of these, 519 and 1847 features with high reproducibility and stability (ICC > 0.80), respectively, were subsequently combined and analyzed using LASSO logistic regression. Finally, 4 handcrafted and 45 deep learning features with nonzero coefficients in LASSO were selected to derive the DLR signature (Supplementary Fig. 1). A detailed description of the selected features is provided in Supplementary Table 1. The signature achieved the AUCs of 0.886 (95% CI, 0.815–0.958), 0.854 (95% CI, 0.778–0.931), and 0.917 (95% CI, 0.854–0.980) in the three EVCs, respectively. The signature was significantly higher in the metastasis group than in the non-metastasis group in all cohorts (*P* < 0.001) (Supplementary Fig. 2). The accuracy, sensitivity, specificity, PPV, and NPV of the signature are presented in Table 2.

**Table 2:** Performance summary of radiologists and different models for prediction of ALN metastasis

Methods		AUC	ACC(%)	SENS(%)	SPE(%)	PPV(%)	NPV(%)	*P* value
Pooled Radiologists		0.703 (0.672-0.735)	72 (69-75)	63 (58-69)	77 (74-81)	62 (57-67)	78 (74-82)	<0.001*
EVC1	CLI	0.769 (0.677-0.860)	75 (66-83)	53 (38-68)	90 (80-96)	77 (59-90)	74 (63-83)	<0.001*
	Signature	0.886 (0.815-0.958)	87 (79-92)	87 (73-95)	87 (76-94)	81 (67-91)	91 (81-96)	0.169
	DLRN	0.914 (0.858-0.971)	88 (80-93)	87 (73-95)	88 (78-95)	83 (69-92)	91 (81-97)	
EVC2	CLI	0.783 (0.687-0.880)	75 (64-83)	69 (51-83)	79 (65-89)	69 (51-83)	79 (65-89)	<0.001*
	Signature	0.854 (0.778-0.931)	72 (62-81)	100 (90-100)	54 (39-68)	59 (46-72)	100 (88-100)	0.011*
	DLRN	0.929 (0.877-0.980)	87 (79-94)	86 (70-95)	88 (77-96)	83 (67-94)	90 (79-97)	
EVC3	CLI	0.700 (0.570-0.830)	68 (55-79)	62 (44-79)	74 (55-88)	71 (51-87)	66 (48-81)	<0.001*
	Signature	0.917 (0.854-0.980)	83 (71-91)	91 (75-98)	74 (55-88)	78 (62-90)	88 (70-98)	0.188
	DLRN	0.952 (0.906-0.997)	89 (78-95)	81 (64-93)	97 (83-100)	96 (81-100)	83 (67-94)	

*EVC* external validation cohort, *CLI* clinical model, *DLRN* deep learning radiomic nomogram, *AUC* the area under the receiver operating characteristic curve, *ACC* accuracy, *SEN* sensitivity, *SPE* specificity, *PPV* positive prediction value, *NPV* negative prediction value.

Statistical quantifications were demonstrated with 95% confidential interval (CI), when applicable. The *P* value indicates the comparison between AUCs of each method and the integrated DLRN by the DeLong test in different cohorts.

### DLR nomogram construction and performance evaluation

**Fig. 1:**
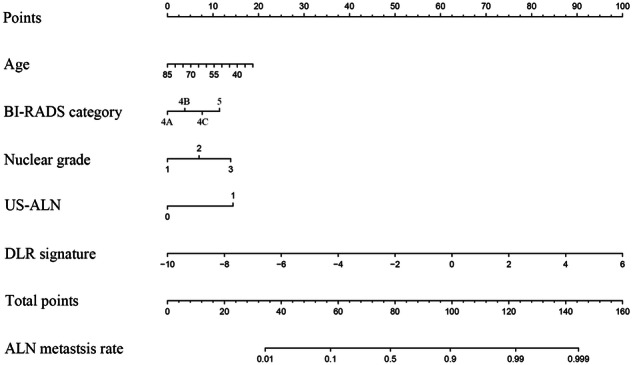
Deep learning radiomics nomogram. Integrated nomogram for axillary lymph node metastasis prediction combined with deep learning radiomics signature and clinicopathological factors. US-ALN, axillary lymph node status reported by axillary ultrasound; DLR deep learning radiomics, ALN axillary lymph node.

The result of univariate logistic regression analysis of the clinical parameters is presented in Supplementary Table 2. The clinical model was developed based on age, BI-RADS category, nuclear grade, and US-ALN by multivariate logistic regression (Table 3). These four independent clinical parameters were significantly correlated with ALN metastasis. The DLRN combined with the DLR signature and independent clinical parameters is shown in Fig. 1. The optimal cutoff of the ALN metastatic rate for DLRN was 0.407, based on the TC.

**Table 3:** Multivariate logistic regression analysis of ALN status in the training cohort

Characteristics	Coefficient	Odds ratio (95% CI)	*P* value
Age	−0.0184	0.9818 (0.9663-0.9972)	0.002*
BI-RADS category	0.3811	1.4638 (1.2499-1.7194)	<0.001*
Nuclear grade	0.3896	1.4764 (1.1394-1.9230)	0.003*
US-ALN	0.7001	2.0140 (1.4050-2.8901)	<0.001*

*CI* confidence interval, *US-ALN* axillary lymph nodes status reported by axillary ultrasound.

**Fig. 2:**
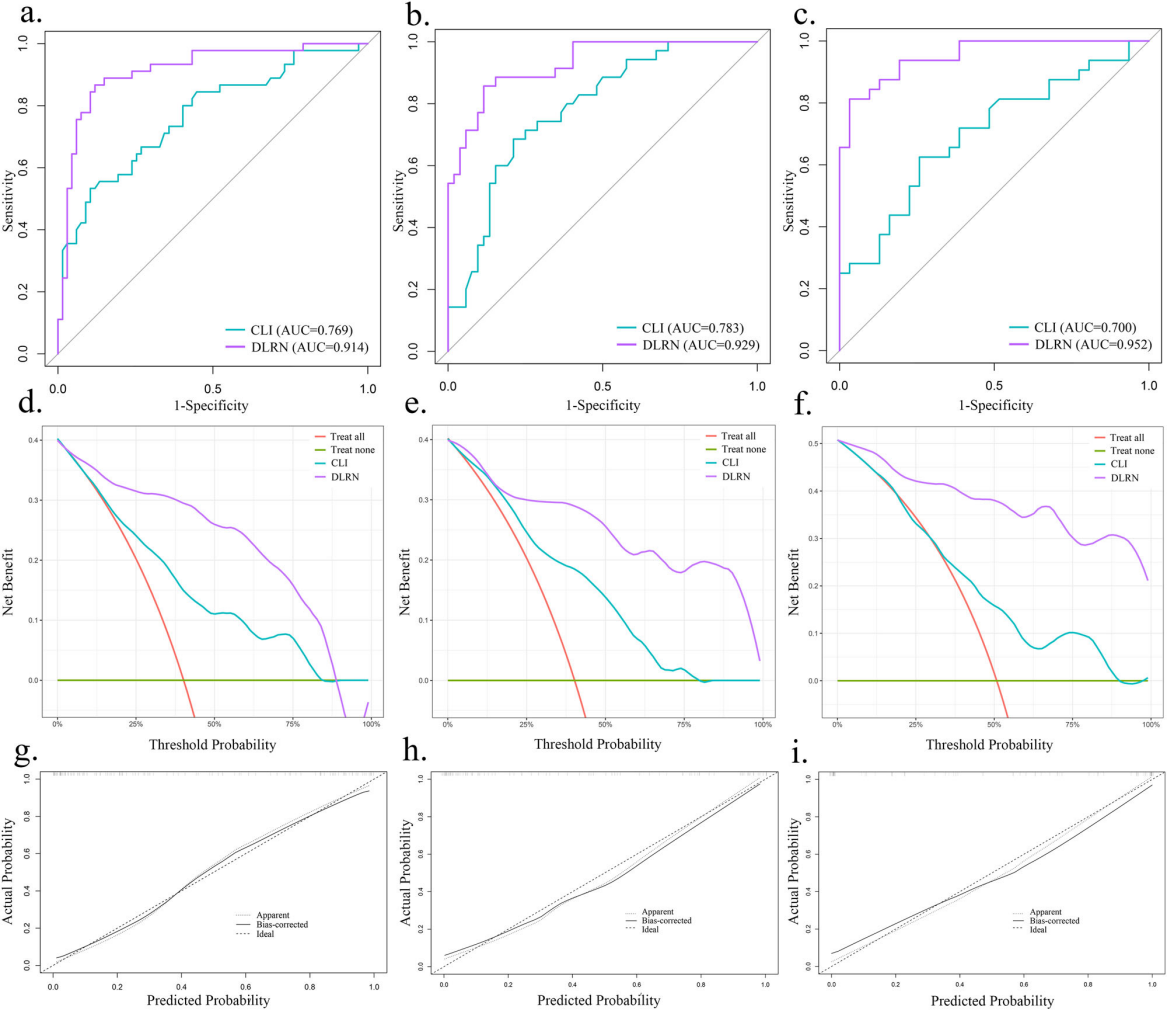
Model performance evaluation. **a**–**c** Receiver operating characteristic area under the curves for the proposed clinical model and DLRN in EVC1, EVC2, and EVC3, respectively. **d**–**f** Decision curves of the clinical model and DLRN in EVC1, EVC2, and EVC3, respectively. **g**–**i** Calibration curves of DLRN in EVC1, EVC2, and EVC3, respectively. EVC, external validation cohort; CLI, clinical model; DLRN, deep learning radiomics nomogram. Note: In Fig. 2d–f, the purple and blue lines represent the DLRN and clinical model, respectively. The orange line represents the assumption that all cases underwent ALN dissection or SLN biopsy. The green line represents the assumption that no cases underwent ALN dissection or SLN biopsy. The decision curve reveals that the DLRN exhibits better performance than the clinical model over a wide range of threshold probabilities.

The DLRN demonstrated significantly better predictive performance than the corresponding clinical model, with AUCs of 0.914 (95% CI, 0.858–0.971), 0.929 (95% CI, 0.877–0.980), and 0.952 (95% CI, 0.906–0.997) in the EVCs (DeLong *P* < 0.001). The AUCs of the DLRN in different surrogate molecular subtypes are presented in Supplementary Table 3. The accuracy, sensitivity, specificity, PPV, and NPV of the clinical model, DLR signature, and DLRN are presented in Table 2. The confusion matrices of the DLRN across all cohorts are shown in Supplementary Fig. 3, and the ROC curves demonstrating the comparative results of the AUCs are displayed in Fig. 2a–c.

**Fig. 3:**
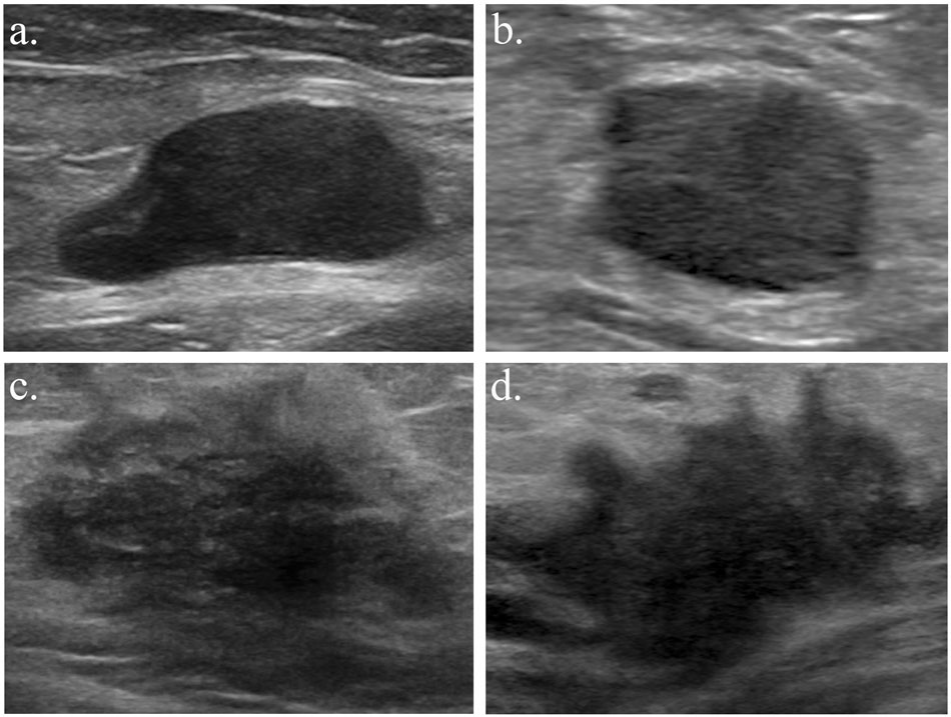
Breast and axillary ultrasonography of two typical cases. **a** Breast ultrasound and **b** axillary ultrasound of a patient with positive ALN metastasis. The patient was wrongly considered as non-metastatic by the radiologists because there were no suspicious ALNs on axillary ultrasound. In addition, benign characteristics of the primary lesion, including a relatively oval shape, smooth margin, homogeneous hypoechoic matrix, and parallel orientation, added the diagnostic difficulty. However, this case was accurately identified as positive ALN metastasis by the DLRN, with a metastatic possibility of 87%. **c** Breast ultrasound and **d** axillary ultrasound of a patient with negative ALN metastasis. The patient was wrongly considered as metastatic type by the radiologists based on the malignant features of the ALN (cortical thickening > 3 mm and microcalcification) and the primary lesion (irregular shape, indistinct margin, heterogeneous echo pattern, and vertical orientation). However, the case was correctly evaluated by the DLRN, with a metastatic possibility of 27%. ALN axillary lymph node, DLRN deep learning radiomics nomogram.

In all the EVCs, 25.3% (38/150) of the non-metastatic ALNs had at least one suspicious feature on ultrasound imaging, and 22.7% (34/150) were misdiagnosed as malignant ALNs by experienced radiologists. Nonetheless, 79.4% (27/34) of them were correctly classified as negative ALNs by the DLRN. A detailed comparison of performance of the DLRN and radiologists is presented in Table 2. The result of ALN status assessment by each radiologist is summarized in Supplementary Table 4, and the typical cases evaluated by human experts and the DLRN are shown in Fig. 3.

The IDI, NRI, and C-index indicated superior classification accuracy of the DLRN compared with the clinical model and DLR signature (Supplementary Table 5). Decision curve analyses (Fig. 2d–f) demonstrated that the DLRN provided a higher net benefit than the clinical model over a wide range of threshold probability. The calibration curves verified that the predicted ALN status by the DLRN was in good agreement with the actual status (Fig. 2g–i). Additionally, a total of 37 patients were included for reproducibility evaluation of the DLRN. The clinical parameters of the 37 patients are presented in Supplementary Table 6. The inter-observer ICC among the three doctors was 0.82, indicating good reproducibility of the DLRN according to Cicchetti’s guideline.

## Discussion

Currently, ALN dissection and SLN biopsy are the standard methods for ALN staging. However, both operations carry varying degrees of postoperative complications and morbidities. Recent researches have concentrated on minimizing unnecessary axillary procedures and avoiding overtreatment for breast cancer. The American Society of Clinical Oncology (ASCO)^31^ has recommended that SLN biopsy can be omitted for clinically node-negative women aged ≥70 with early-stage invasive breast cancer, that is HER2-negative and hormone receptor-positive. The Sentinel Node vs Observation After Axillary Ultra-Sound (SOUND) trial^32^ concluded that SLN biopsy can be safely spared in patients with small breast cancer (diameter≤2 cm) and a negative result on axillary ultrasonography. Additionally, several ongoing clinical trials are exploring the possibility of SLN biopsy omission for early breast cancer patients receiving neoadjuvant systemic therapy^33^ or breast-conserving surgery^34^. In our study, we successfully developed a DLRN to assess ALN status preoperatively for breast cancer. The DLRN exhibited satisfactory performance in all EVCs, with AUCs of 0.914, 0.929, and 0.952 and accuracies of 88%, 87%, and 89% in EVC1, EVC2, and EVC3, respectively. This represents a promising approach to predict ALN status and avoid unnecessary axillary treatment.

Among the three EVCs, 22.7% (34/150) of the non-metastatic ALNs were misdiagnosed by the five experienced radiologists. However, 79.4% (27/34) of them were correctly classified by the DLRN. On the other hand, 42.0% (47/112) of the metastatic ALNs showed no suspicious features on axillary ultrasound, which is in accordance with the results of another study^13^. Nonetheless, 72.3% (34/47) of the cases were successfully detected by the DLRN. In addition, the AUC of the DLRN was significantly higher than that of the experienced radiologists (0.665–0.703), consistent with the AUCs of radiologists in other studies (0.585–0.735)^10,12,26^. The false-negative rate of DLRN was also comparable to that of SLN biopsy, ranging from 7.8 to 27.3%^35^. Therefore, for patients with a low risk of ALN metastasis by the nomogram, an observational strategy could be recommended instead of invasive axillary treatment. For patients with a high risk of ALN metastasis, axillary operation might be required during management.

Previous studies reported various clinical models for predicting ALN metastasis. Isaac et al. ^36^ developed a promising multiparametric score to assess the status of nonsentinel lymph nodes in clinically node-negative breast cancer with positive SLNs after systemic therapy. In addition, a representative Memorial Sloan Kettering Cancer Center nomogram^14^ was developed and commonly used in different populations. Nevertheless, certain variables in this nomogram, such as histological tumor size and lymphovascular invasion, could only be obtained postoperatively, potentially impeding its clinical applicability. In this study, all the factors included in the DLRN can be achieved preoperatively, and the predictive performance was satisfactory when validated in three external hospitals.

Various scholars have explored the additional value of DLR in predicting ALN metastasis. Ding et al. ^37^ developed a deep learning model based on core needle biopsy specimen to identify ALN status. However, the model performance was expected to be further enhanced (AUC = 0.725) when validated externally. Moreover, biopsy specimens were incapable of capturing the heterogeneity of the entire tumor, and the acquisition of specimens was largely operator-dependent. Consequently, an evaluation of model reproducibility is warranted. Zheng et al. ^26^ developed a DLR model based on ultrasound and shear wave elastography for breast cancer. The DLR model could predict ALN status (N0 vs. N+[≥1]) and discriminate metastatic burden (N+[1, 2] vs. N+[≥3]) of ALNs with favorable performance. However, the model faced limitation in clinical application due to the nonroutine use of elastography. Compared with the studies, our DLRN was constructed based on a large sample size and with good reproducibility verified by a prospective trial. In addition, to relieve overfitting^38^ – a common pitfall that may constrain the clinical utility of deep learning models, we initially incorporated handcrafted features to complement DLR features and then utilized LASSO to reduce feature dimensionality. This approach could effectively minimize the risk of overfitting caused by excessive features. The stable and good performance of the DLRN in three external hospitals demonstrated that model overfitting had been mitigated in this study.

In this study, the DLR signature comprised 4 CR and 45 DLR features, which were significantly correlated with ALN status. The four key CR features were local binary pattern (LBP) features, characterized as straightforward, resilient, and efficient texture descriptors^39^. Some studies have reported that LBP features could promote faster diagnosis of breast malignancies imaged by shear wave elastography^40^ and the classification of various types of breast lesions based on optical coherence microscopy^41^. According to these findings, LBP features may reflect different patterns of heterogeneity of breast masses, which may be involved in the occurrence of ALN metastasis.

In the clinical model, young age, high BI-RADS category, high nuclear grade, and positive US-ALN were associated with increased risk of ALN metastasis. The relationship between these variables and ALN status has been confirmed in earlier studies^10,14,42^. However, these correlations were not consistent across all the studies, and the results were not stable for the EVCs in our study. For example, multivariate analysis in the TC identified age as an independent predictor of ALN status (*P* = 0.002). However, the correlation was not robust in the EVCs (*P* = 0.860 for EVC1, 0.737 for EVC2, and 0.287 for EVC3). On the other hand, some studies have identified tumor size, tumor classification, Ki-67, ER, and PR status as independent predictive factors of metastatic ALNs in breast cancer^14,43^. However, these findings were not observed in the present study. The reason for these inconsistent results may be that clinical parameters only reflect limited aspects of the lesions. Therefore, we integrated the DLR signature with clinical parameters to construct the DLRN and achieved far better predictive performance than the clinical model (*P* < 0.001) in all cohorts.

Our study still has some limitations. First, inherent variations and shortages were inevitable. For instance, the quality of ultrasonography varied because the operations were performed by different radiologists. Second, the two-dimensional image cannot represent the entire tumor and the information in three-dimensional lesions might be missed. Third, our study exclusively included patients with unifocal breast lesions because it was difficult to identify the lesion responsible for ALN metastasis among multifocal and multicentric lesions. Non-mass-type lesions were also excluded in our study due to the difficulty of ROI segmentation. Fourth, although various researchers have identified the association between different molecular subtypes and ALN status, our study encountered limitations in constructing models for each subtype individually, given the restricted sample size for each subtype. Therefore, further improvements must be made with a more comprehensive analysis in the future.

In summary, we established a deep learning radiomics nomogram to preoperatively evaluate axillary lymph node status in patients with unifocal breast cancer. The nomogram outperformed both the clinical model and radiologists. Therefore, with favorable specificity and sensitivity, this model can offer a potential non-invasive approach to identify lymph node metastasis and guide clinical decision making.

## Methods

### Patients

This study was approved by the institutional review board of Nanjing Drum Tower Hospital (approval no. 202214201) and compliant with the ethics standards of the regulations of the Declaration of Helsinki. The requirement for informed consent was waived owing to the retrospective nature in this study.

The inclusion criteria were listed as follows: (a) women with histologically diagnosed unifocal breast cancer, (b) patients with confirmed ALN status by ALN dissection/SLN biopsy, and (c) patients received ultrasound examination within 1 week before surgery. The exclusion criteria were as follows: (a) patients received neoadjuvant radiotherapy, chemotherapy, or other therapies preoperatively, (b) patients with ultrasound-invisible or non-mass-type lesions, (c) patients with multifocal lesions or insufficient image quality, (d) patients with metastatic breast cancer, and (e) patients with incomplete clinical or histopathological information. Noteworthily, multifocal lesions were excluded due to the difficulty of distinguishing the responsible lesion which caused metastasis from various masses.

**Fig. 4:**
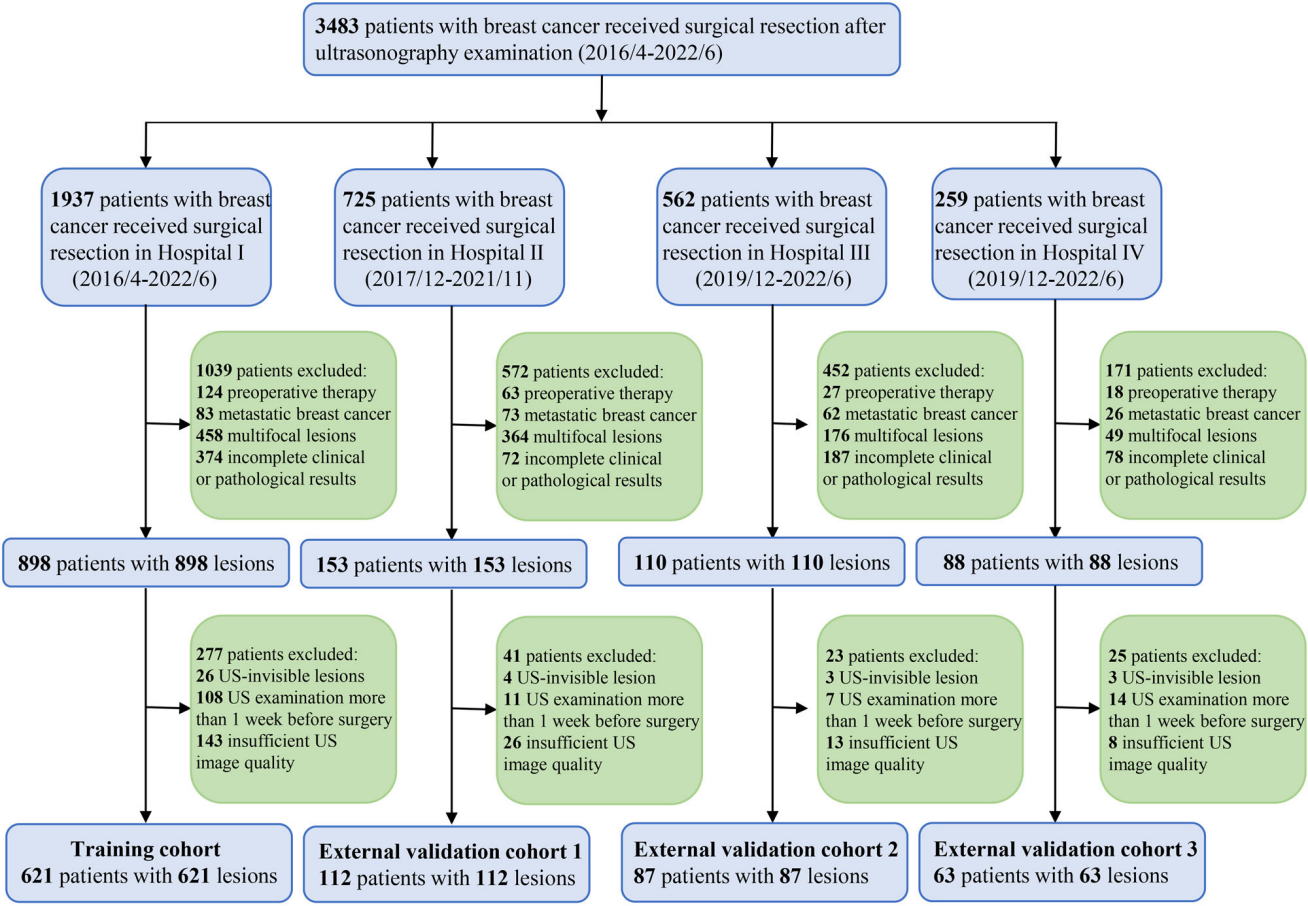
Flow diagram of the study population. In total, 883 of the 1530 patients with breast cancer were included according to the selection criteria.

In total, 883 patients with histologically confirmed primary breast cancer from four hospitals were included in this study. A flowchart of the patient recruitment process is shown in Fig. 4. Finally, 621 patients with breast cancer from Hospital I (Nanjing Drum Tower Hospital) between April 1, 2016, and June 30, 2022, were reviewed and identified as the training cohort (TC). From December 30, 2017, to November 1, 2021, 112 patients from Hospital II (Jinling Hospital) were recruited as EVC1. From December 1, 2019, to June 30, 2022, 87 patients from Hospital III (Jiangbei Hospital) and 63 patients from Hospital IV (Taizhou Hospital) were enrolled as two EVCs (EVC2 and EVC3).

### Clinical parameters

The preoperative clinical parameters collected for analysis included clinicopathological characteristics and ultrasound findings of the breast and axilla. Clinicopathological characteristics included age, body mass index (BMI), estrogen receptor (ER) status, progesterone receptor (PR) status, human epidermal growth factor receptor 2 (HER2) expression, Ki-67 expression, nuclear grade, tumor classification, and surrogate subtype^44^. The pathological characteristics were obtained from needle biopsy, which is the standard preoperative procedure for breast cancer. According to the 2017 St Gallen International Expert Consensus^45^, ER-positive status was identified when ER-positive rate ≥1%, and PR-positive status was identified when PR-positive rate ≥1%. HER2 positivity was identified by an immunohistochemical score of 3+ or a score of 2+ with gene amplification. Cases failing to meet the criteria were classified as HER2-negative. In terms of Ki-67, cases with more than 14% positive nuclei were categorized as high Ki-67 expression, whereas others were classified as low Ki-67 expression.

The ultrasound findings of the breast and axilla included tumor location, ultrasound size of the breast lesion, Breast Imaging Reporting and Data System (BI-RADS) category, and ALN status reported by axillary ultrasound (US-ALN). On axillary ultrasound, suspicious metastatic ALNs were identified if any of the following features were present: (1) longest/shortest axis ratio < 2, (2) cortical thickening > 3 mm, or (3) loss of the fatty hilum in the node. Non-suspicious ALNs were identified when no suspicious features were found. The BI-RADS category and US-ALN were evaluated by two experienced radiologists (B.J. and X.J., with 8 and 9 years of breast US experience, respectively) blinded to pathological results. The inter-/intra-observer agreement of BI-RADS category and US-ALN was evaluated using the Kappa test (detailed in Supplementary Method 1). Landis and Koch’s evaluation^46^ was utilized to interpret the Kappa value.

### Ultrasonography examination and image preprocessing

According to the Guidelines of the American Institute of Ultrasound in Medicine practice^47^, ten radiologists with at least 5 years of breast ultrasound experience from the four hospitals performed preoperative breast and axillary ultrasound. Patients were kept in the supine position, and the field of view was set to contain the pectoralis muscle at the deepest aspect of breast ultrasound. The detailed equipment for the ultrasound examination is listed in Supplementary Table 7, and the procedures of ultrasound examination are detailed in Supplementary Method 2. For each patient, one single image of the target breast mass was selected at the maximum diameter plane for further analysis.

To compare the performance of ALN status assessment between radiologists and the model, five radiologists with more than 10 years of experience evaluated the ALN status according to breast and axillary ultrasound examinations. Every radiologist assessed the ALN status of all patients independently and was blinded to histopathological status. The consensus or prevailing viewpoint of the five radiologists served as the result of human experts.

The region of interest (ROI) of the primary breast lesion for each ultrasound image was segmented by reader 1 (W.T., with over 15 years of breast US interpretation experience) using the ImageJ software (http://imagej.net). One month later, 60 random patients were selected and delineated again by readers 1 and 2 (H.Y., with 8 years of breast US interpretation experience). The inter- and intra-observer reproducibility of tumor segmentation and DLR/CR feature extraction were analyzed using breast ultrasound in 60 randomly selected patients for ROI-based feature extraction in a blinded manner by the two readers. An inter-/intra-class correlation coefficient (ICC) of the features > 0.80 indicates good agreement with the tumor segmentation and feature extraction, according to Cicchetti’s guidelines^48^. Based on the ROI of each lesion, the top, bottom, left, and right boundary points were automatically generated to create the bounding box. The rectangular bounding box was then cropped from the original image, resized to 224 × 224 pixels, normalized, and fed into the convolutional neural network as an input layer.

### Deep learning radiomics (DLR) feature extraction and signature construction

**Fig. 5:**
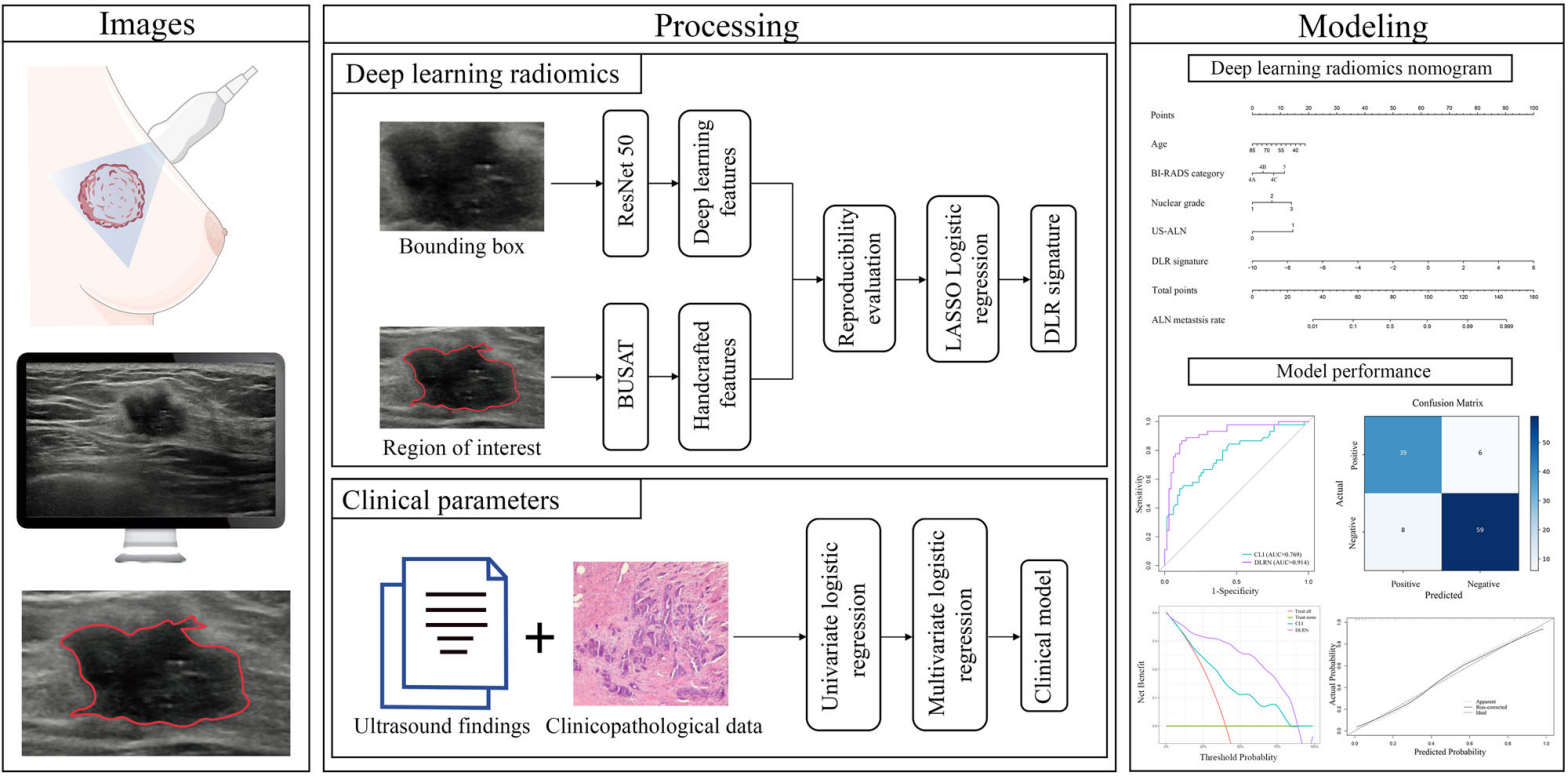
Flowchart of deep learning radiomics nomogram construction. Workflow of deep learning radiomics nomogram construction for predicting axillary lymph node metastasis in patients with breast cancer. BUSAT Breast Ultrasound Analysis Toolbox; LASSO least absolute shrinkage and selection operation; DLR, deep learning radiomics.

A flowchart of the study is shown in Fig. 5. Handcrafted features including textural and BI-RADS features were extracted in MATLAB 2021b using the Breast Ultrasound Analysis Toolbox^49^. Deep learning features were extracted using ResNet50^50^ (Supplementary Fig. 4) and the detailed procedure was presented in Supplementary Method 3. In brief, the fully connected layer and softmax layer of ResNet50 were removed, and the output values of the nodes in last layer were identified as the deep learning features. Subsequently, the handcrafted and deep learning features were combined. The least absolute shrinkage and selection operator (LASSO) logistic regression algorithm^51^ was used to select the key features related to ALN status and compile the DLR signature.

### DLR nomogram construction

The clinical parameters were integrated into the DLR model to improve predictive performance. In the TC, univariate logistic regression analysis was used to identify candidate factors among the clinical parameters. Furthermore, multivariate logistic regression was employed to select independent clinical parameters and construct a clinical model. We then integrated the DLR signature and independent clinical parameters using multivariate logistic regression to construct a combination model. The combination model was finally converted into an individualized DLRN.

### Model performance

Integrated discrimination improvement (IDI), net reclassification improvement (NRI), and C-index were used to demonstrate the prediction ability. ROC curve analysis and the AUC with a 95% confidence interval (CI) were used for interpretation. AUCs were compared using the DeLong test^52^. The AUCs of the DLRN in predicting ALN metastasis for different surrogate molecular subtypes were also calculated. The optimal cutoff value of the DLRN was determined using the Youden index of the TC. Boxplots and confusion matrices were used to visualize the performance of the DLR signature and DLRN, respectively. The accuracy, sensitivity, specificity, positive predictive value (PPV), and negative predictive value (NPV) with 95% CIs were also evaluated. Decision curve and calibration curve analyses were performed to assess the clinical value and calibration of each model, respectively.

Reproducibility of the DLRN was assessed in 37 patients who were prospectively enrolled from Hospital I between January 8, 2024, and January 19, 2024. The workflows of the patient recruitment and reproducibility evaluation are shown in Supplementary Fig. 5. Three doctors with varying experience in breast ultrasound (3, 7, and 12 years, respectively) independently performed predictive procedures, including imaging acquisition, ROI segmentation, feature extraction, DLR signature acquisition, clinical data input, and probability calculation for the same lesion in each patient. The inter-observer ICC was calculated to assess model reproducibility.

### Statistical analysis

Two-tailed *P* < 0.05 denoted a significant difference. All statistical analyses were conducted in R 4.1.2, Python 3.6, and MATLAB R2021b. Differences in continuous data were compared using the independent sample t-test or Mann–Whitney exact U test. Categorical variables were compared using the chi-squared or Fisher’s exact test. The code of predicting procedure can be available in https://github.com/ZouLiwen-1999/ALN_metastasis_Pred.

### Reporting summary

Further information on research design is available in the Nature Research Reporting Summary linked to this article.

### Supplementary information

Supplementary material

Reporting summary

## Data Availability

Access to the original images and clinical data in this study are available from the corresponding author on reasonable request.

## Code availability

The code for prediction of lymph node metastasis is publicly available in https://github.com/ZouLiwen-1999/ALN_metastasis_Pred.
