# Supplementary material for: Deep learning radiomics based prediction of axillary lymph node metastasis in breast cancer
Source: NPJ Breast Cancer. 2024 Mar 12;10:22. doi: 10.1038/s41523-024-00628-4 (PMC10933422; doi:10.1038/s41523-024-00628-4)

Supplementary Material

Supplementary Notes: Significant differences in clinical parameters between the training cohort and validation cohorts

In terms of clinical parameters, BI-RADS category was lower in the training cohort (TC) than in the external validation cohort 1 and 2 (EVC1 and EVC2) ($P < 0.001$). In the TC, most of the tumors located in the central quadrant; however, in the EVC3, approximately half of the tumors located in the upper outer quadrant. More patients in the TC had positive ER status than in the EVC3 (72.6% vs. 55.6%, $P < 0.001$) and more patients in the TC had positive HER-2 status than in the EVC1 (83.7% vs. 30.4%, $P < 0.001$). In addition, significant differences in molecular subtype were observed between the TC and three EVCs. In the TC, more patients with Luminal A were observed than in the EVCs (39.9% vs. 14.3% vs. 6.9% vs. 1.6%, $P < 0.001$), and less patients in the TC had Luminal B subtype than in the EVCs (34.8% vs. 63.4% vs. 66.7% vs. 61.9%, $P < 0.001$).

Supplementary Method 1 Inter- and intra-observer agreement of BI-RADS category and US-ALN

To evaluate the intra-observer agreement for BI-RADS category and ALN status reported by axillary ultrasound (US-ALN), radiologist 1 (P.W., with 8 years of breast ultrasound experience) repeated evaluating the random selected 30 patients at a time interval of 1 month. Inter-observer agreement was tested by radiologist 1 and 2 (X.J., with 9 years of breast ultrasound experience), evaluating the same ALNs independently in another 30 patients. Kappa test was used to evaluate the intra- and inter-observer agreement. Landis and Koch's evaluation (absent, <0.10; weak, 0.10–0.20; fair, 0.21–0.40; moderate, 0.41–0.60; substantial, 0.61–0.80; nearly perfect, 0.81–1.00) was utilized to interpret the Kappa value.

Supplementary Method 2 Procedure of breast and axillary ultrasonography

When performing breast and axillary ultrasonography, the patients were kept in supine position, with the ipsilateral arms elevated at least 90 degrees and the corresponding hands placed behind the heads. This allowed a comfortable and seamless examination of the breast and axilla. Application pressure were selected so that the anatomy could be visualized on the B-mode image without artifacts and the amount of pressure applied to the breast was not uncomfortable for patients. Transverse/sagittal/radial scan orientation were used when scanning the breast. In practice, the examiner decided the scan orientations to make sure that the entire breast was scanned and visualized in an overlapping manner. All of the images were stored in the Picture Archiving and Communication Systems for further research.

Supplementary Method 3 Handcrafted feature and deep learning feature extraction methodology

Handcrafted features were extracted by using the function "Preprocessing_and_FeatureExtraction" in the Breast Ultrasound Analysis Toolbox[4]. We extracted 544 handcrafted features for each ROI. The detailed information of these features was available in the documentation for the toolbox (DOI:10.1007/978-3-319-59226-8_26).

According to Zheng's research[5] which incorporated ultrasound and elastography images to develop DLR model, ResNet50[6] achieved the best performance among ResNet101, Inception V3,

and VGG19 in predicting ALN status. So ResNet50 was adopted as the base model to extract the deep learning features in our study. Transfer learning was utilized to help fine-tune all weights and biases and reduce training time significantly. In our model, parameters pretrained on the ImageNet dataset were used and after loading that, we used our dataset for retraining. At last, the original classifier for the ImageNet classes was replaced by a binary classifier so that the output was a class probability vector ranging from 0 to 1 as the prediction result for each patient. The network was trained from scratch with cross entropy loss function and Adam optimizer with a learning rate of 0.0001 and a batch size of 64. Data augmentation was applied for the training cohort to reduce the potential bias caused by the limited number of images in the training procedure[7]. Ultrasound images in the training cohort were augmented through several random transformations, including image flipping horizontally and vertically, cropping, and rotation[8], which could increase the training data pool and decrease the overfitting of the generated DLR model. In the ResNet50 model, the fully connected layer and softmax layer were removed, and the output value of the nodes in last layer was used as the deep learning features. Ultimately, the ResNet50 model extracted 2048 deep learning features from each breast ultrasound image.

Supplementary References

1. Curigliano, G. *et al.* De-escalating and escalating treatments for early-stage breast cancer: The St. Gallen International Expert Consensus Conference on the Primary Therapy of Early Breast Cancer 2017. *Annals of Oncology* **28**, (2017).
2. Goldhirsch, A. *et al.* Strategies for subtypes-dealing with the diversity of breast cancer: Highlights of the St Gallen international expert consensus on the primary therapy of early breast cancer 2011. *Annals of Oncology* **22**, (2011).
3. Tan, P. H. *et al.* The 2019 World Health Organization classification of tumours of the breast. *Histopathology* vol. 77 Preprint at https://doi.org/10.1111/his.14091 (2020).
4. Rodríguez-Cristerna, A., Gómez-Flores, W. & de Albuquerque-Pereira, W. C. BUSAT: A MATLAB toolbox for breast ultrasound image analysis. in *Lecture Notes in Computer Science (including subseries Lecture Notes in Artificial Intelligence and Lecture Notes in Bioinformatics)* vol. 10267 LNCS (2017).
5. Zheng, X. *et al.* Deep learning radiomics can predict axillary lymph node status in early-stage breast cancer. *Nat Commun* **11**, (2020).
6. He, K., Zhang, X., Ren, S. & Sun, J. Deep residual learning for image recognition. in *Proceedings of the IEEE Computer Society Conference on Computer Vision and Pattern Recognition* vols 2016-December (2016).
7. Chawla, N. v., Bowyer, K. W., Hall, L. O. & Kegelmeyer, W. P. SMOTE: Synthetic minority over-sampling technique. *Journal of Artificial Intelligence Research* **16**, (2002).
8. Lee, Y. W., Huang, C. S., Shih, C. C. & Chang, R. F. Axillary lymph node metastasis status prediction of early-stage breast cancer using convolutional neural networks. *Comput Biol Med* **130**, (2021).

Supplementary Table 1 Description of LASSO picked radiomics features

Handcrafted features			
Selected Features	Coefficient	Category	Feature description
LBP_38	-261.053	Texture-LBP	Local binary pattern features, mode:38
LBP_172	1730.081	Texture-LBP	Local binary pattern features, mode:172
LBP_198	3537.878	Texture-LBP	Local binary pattern features, mode:198
LBP_206	-787.685	Texture-LBP	Local binary pattern features, mode:206

Deep learning features			
Selected Features	Coefficient	Selected Features	Coefficient
f_135	-0.441	f_1037	1.252
f_252	-0.208	f_1076	-1.160
f_272	-0.945	f_1144	-0.024
f_311	-2.122	f_1145	-1.222
f_374	-1.350	f_1170	0.099
f_375	0.119	f_1222	0.764
f_384	-0.115	f_1304	1.423
f_400	-0.019	f_1313	1.034
f_457	-0.120	f_1417	-2.191
f_542	0.440	f_1494	0.957
f_558	0.503	f_1506	-0.309
f_644	0.452	f_1523	-0.551
f_700	-0.155	f_1556	-0.649
f_762	0.097	f_1557	-0.129
f_779	0.213	f_1606	-0.304
f_782	-0.104	f_1612	0.829
f_802	0.434	f_1644	0.126
f_835	-0.712	f_1802	0.297
f_883	0.166	f_1874	-0.126
f_895	0.020	f_1891	-0.155
f_912	-0.410	f_1995	-0.222
f_1028	-0.312	f_2033	0.101
f_1036	0.071		

Supplementary Table 2 Univariate logistic regression analysis of ALN metastasis in the training cohort

Characteristic	Coefficient	Odds ratio (95% CI)	P value
Age	-0.0168	0.9833(0.9689-0.9977)	0.024*
BMI	0.1546	1.3461(1.2450-1.5612)	0.274
BI-RADS category	0.4355	1.5457(1.3335-1.7978)	<0.001*
Location	-0.0680	0.9343(0.8391-1.0406)	0.215
Ultrasound size	0.2245	1.2518(1.0739-1.4629)	0.004*
Nuclear grade	0.4092	1.5056(1.1779-1.9344)	0.001*
Tumor classification	0.0555	1.0571(0.7988-1.3920)	0.694
ER	-0.0763	0.9265(0.6422-1.3443)	0.685
PR	-0.0677	0.9345(0.6627-1.3221)	0.701
HER2	0.4395	1.5520(0.9754-2.5336)	0.070
Ki-67	0.4221	1.5252(0.9707-2.4546)	0.074
Surrogate subtype	0.1622	1.1761(0.9772-1.4153)	0.086
US-ALN	0.9570	2.6038(1.8545-3.6680)	<0.001*

Abbreviations: CI, confidence interval; BMI, body mass index; ER, estrogen receptor; PR, progesterone receptor; HER-2, human epidermal growth factor receptor-2; US-ALN, axillary lymph nodes status reported by axillary ultrasound.

Supplementary Table 3 Model performances in different surrogate subtypes

Surrogate subtype	EVC1		EVC2		EVC3	
	AUC	95%CI	AUC	95%CI	AUC	95%CI
Luminal A-like	0.982	0.931-1.000	1.000	1.000-1.000	NA	NA
Luminal B-like	0.917	0.846-0.988	0.908	0.832-0.984	0.966	0.918-1.000
HER2-overexpression	1.000	1.000-1.000	0.990	0.961-1.000	0.963	0.876-1.000
Triple negative	0.909	0.731-1.000	1.000	1.000-1.000	0.813	0.425-1.000

Abbreviations: EVC, external validation cohort; AUC, the area under the receiver operating characteristic curve; CI, confidence interval; NA, not appliable.

Note: AUC is not appliable for Luminal A-like subtype in EVC3 because there is only one case in the cohort.

Supplementary Table 4 Axillary lymph nodes assessment by radiologists based on breast and axillary ultrasonography

Radiologists	AUC	ACC(%)	SENS(%)	SPE(%)	PPV(%)	NPV(%)
Reader1	0.665(0.633-0.697)	69(66-72)	57(52-63)	76(72-79)	58(52-63)	75(71-79)
Reader2	0.677(0.645-0.709)	69(66-72)	61(56-66)	74(70-78)	58(53-64)	76(73-80)
Reader3	0.684(0.652-0.715)	70(67-73)	61(56-66)	76(72-79)	60(54-65)	77(73-80)
Reader4	0.685(0.653-0.717)	70(67-73)	63(58-69)	74(70-77)	59(53-64)	77(74-81)
Reader5	0.691(0.659-0.723)	71(68-74)	62(56-67)	76(73-80)	61(55-66)	77(74-81)
Pooled Readers	0.703(0.672-0.735)	72(69-75)	63(58-69)	77(74-81)	62(57-67)	78(74-82)

Abbreviations: AUC, the area under the receiver operating characteristic curve; ACC, accuracy; SEN, sensitivity; SPE, specificity; PPV, positive predictive value; NPV, negative predictive value. Note: Pooled readers' final prediction is identified by the consensus or prevailing viewpoint of the five radiologists. Statistical quantifications were demonstrated with 95% confidence interval (CI).

Supplementary Table 5 Model performances in the validation cohorts.

	EVC1			EVC2			EVC3		
Model	C-index	95%CI		C-index	95%CI		C-index	95%CI	
CLI	0.768	0.751-0.786		0.783	0.763-0.804		0.700	0.668-0.732	
DLRS	0.886	0.873-0.900		0.854	0.838-0.871		0.917	0.902-0.933	
DLRN	0.914	0.904-0.925		0.929	0.918-0.939		0.952	0.940-0.963	
	NRI	95%CI	P value	NRI	95%CI	P value	NRI	95%CI	P value
DLRN VS CLI	1.301	1.017-1.585	<0.001*	1.024	0.655-1.393	<0.001*	1.176	0.776-1.575	<0.001*
DLRS VS CLI	0.632	0.272-0.991	<0.001*	0.451	0.033-0.868	0.034*	1.111	0.700-1.522	<0.001*
	IDI	95%CI	P value	IDI	95%CI	P value	IDI	95%CI	P value
DLRN VS CLI	0.315	0.231-0.399	<0.001*	0.308	0.205-0.412	<0.001*	0.505	0.378-0.632	<0.001*
DLRS VS CLI	0.2085	0.089-0.328	<0.001*	0.157	0.011-0.034	0.036*	0.402	0.244-0.561	<0.001*

Abbreviations: EVC, external validation cohort; CLI, clinical model; DLRS, deep learning radiomics signature; DLRN, deep learning radiomics nomogram; NRI, Net reclassification improvement; IDI, Integrated discrimination improvement; CI, confidence interval.

Note: *P value < 0.05, P values were calculated by NRI test and IDI test.

Supplementary Table 6 Baseline characteristics of patients included for reproducibility evaluation.

Characteristic	Patients (n=37)	Characteristic	Patients (n=37)
Age (years)	54.65±11.36	ER	
Ultrasound size (cm)	2.02±0.85	Positive	19 (51.35%)
BMI		Negative	18 (48.65%)
<25	17 (45.95%)	PR	
25-30	14 (37.84%)	Positive	27 (72.97%)
>30	6 (16.22%)	Negative	10 (27.03%)
Not appliable	0 (0.00%)	HER-2	
BI-RADS category		Positive	29 (78.38%)
4A	9 (24.32%)	Negative	8 (21.62%)
4B	14 (37.84%)	Ki-67	
4C	13 (35.14%)	Positive	28 (75.68%)
5	1 (2.70%)	Negative	9 (24.32%)
Tumor location		Surrogate subtype	
UOQ	18 (48.65%)	Luminal A-like	0 (0.00%)
LOQ	6 (16.22%)	Luminal B-like	25 (67.57%)
UIQ	4 (10.81%)	HER-2-overexpression	9 (24.32%)
LIQ	8 (21.62%)	Triple negative	3 (8.11%)
Central	1 (2.70%)	US-ALN	
Nuclear grade		Suspicious	10 (27.03%)
I	25 (67.57%)	Non-suspicious	27 (72.97%)
II	9 (24.32%)	ALN metastasis	
III	3 (8.11%)	Positive	14 (37.84%)
Tumor classification		Negative	23 (62.16%)
Noninvasive carcinoma	2 (5.41%)		
Invasive carcinoma			
NST	35 (94.59%)		
ST	0 (0.00%)		
Rare carcinoma	0 (0.00%)		

Abbreviations: BMI, body mass index; UOQ, upper outer quadrant; LOQ, lower outer quadrant; UIQ, upper inner quadrant; LIQ, lower inner quadrant; NST, no special type; ST, special type; ER, estrogen receptor; PR, progesterone receptor; HER-2, human epidermal growth factor receptor-2; US-ALN, axillary lymph nodes status reported by axillary ultrasound; ALN, axillary lymph node.

Supplementary Table 7 Equipment for ultrasonography examination in different hospitals

Hospital	Machine	Probe	Number of patients
Hospital I	Siemens S3000	linear transducer operating at 4-9 MHz	365
	Philips IU22	linear transducer operating at 6-12 MHz	178
	Philips EPIQ5	linear transducer operating at 6-12 MHz	45
	GE Healthcare LOGIQ E9	linear transducer operating at 6-15 MHz	33
Hospital II	Siemens S2000	linear transducer operating at 4-9 MHz	87
	GE Healthcare LOGIQ E9	linear transducer operating at 6-15 MHz	25
Hospital III	Philips EPIQ5	linear transducer operating at 6-12 MHz	57
	MyLab Twice	linear transducer operating at 5-13 MHz	30
Hospital IV	Philips IU22	linear transducer operating at 6-12 MHz	63

Supplementary Figure 1 Feature selection by using the least absolute shrinkage and selection operator (LASSO) logistic regression. (A) Tuning parameter lambda selection. Based on the 1 standard error of the minimum criteria, the tuning parameter lambda was selected through 10-fold cross validation using the LASSO model. The relation between mean-squared error and log lambda was plotted and a vertical line was drawn at the optimal value via 1 standard error of the minimum criteria and the minimum criteria. (B) LASSO coefficient distribution of radiomics features.

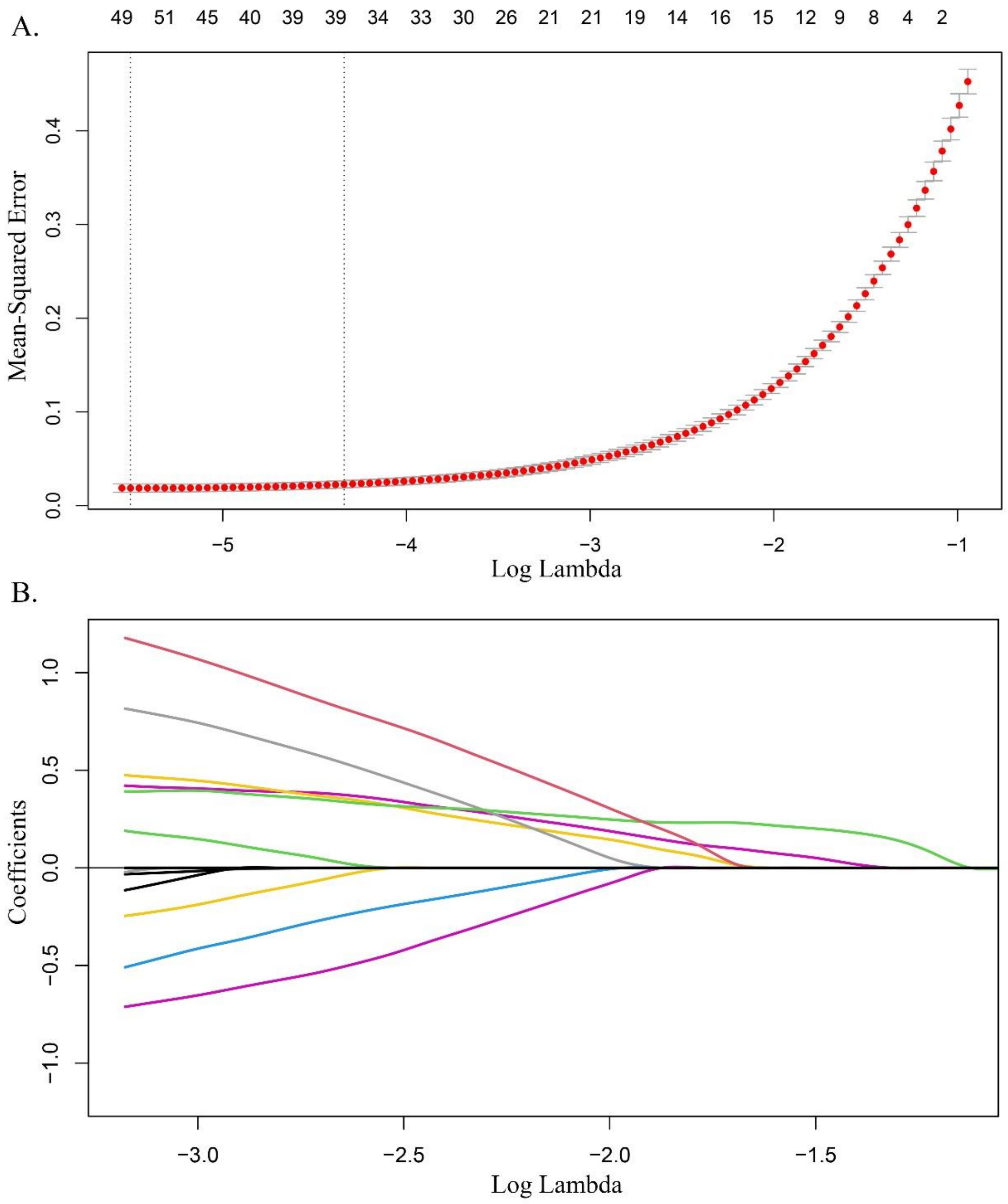

Supplementary Figure 2 Boxplots regarding DLR signature between the metastatic and non-metastatic groups in the TC (A), EVC1 (B), EVC2 (C), and EVC3 (D). DLR, deep learning radiomics; TC, training cohort; EVC, external validation cohort.

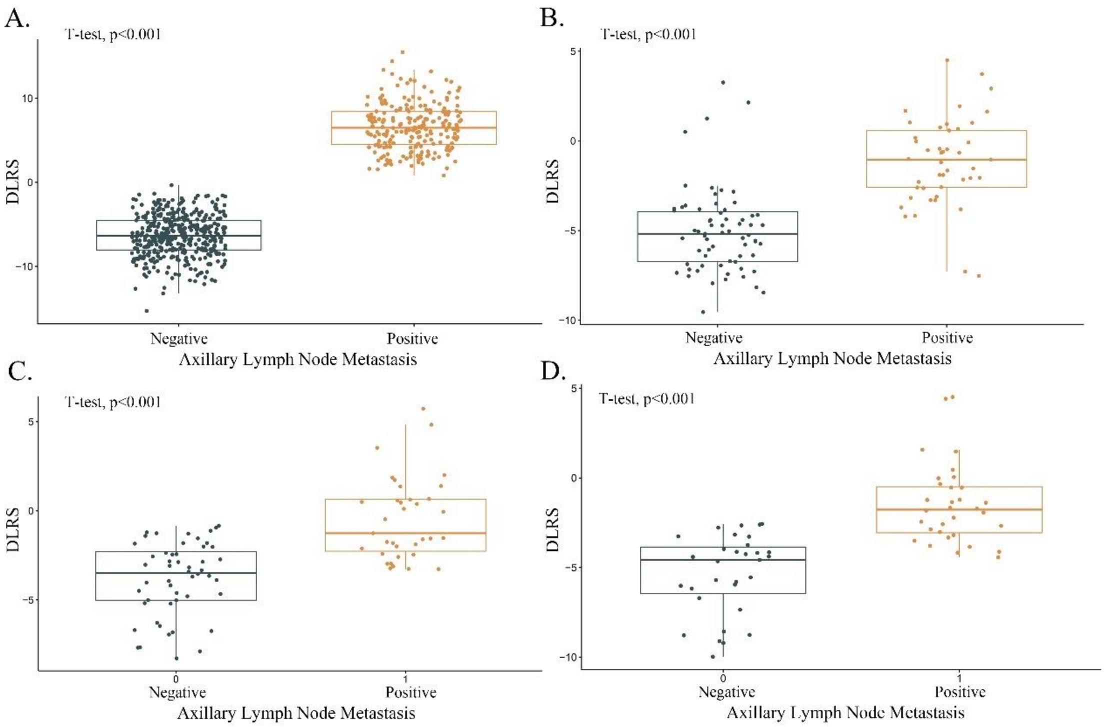

Supplementary Figure 3 Confusion matrices of deep learning radiomics nomogram in the TC (A), EVC1 (B), EVC2 (C), and EVC3 (D). TC, training cohort; EVC, external validation cohort.

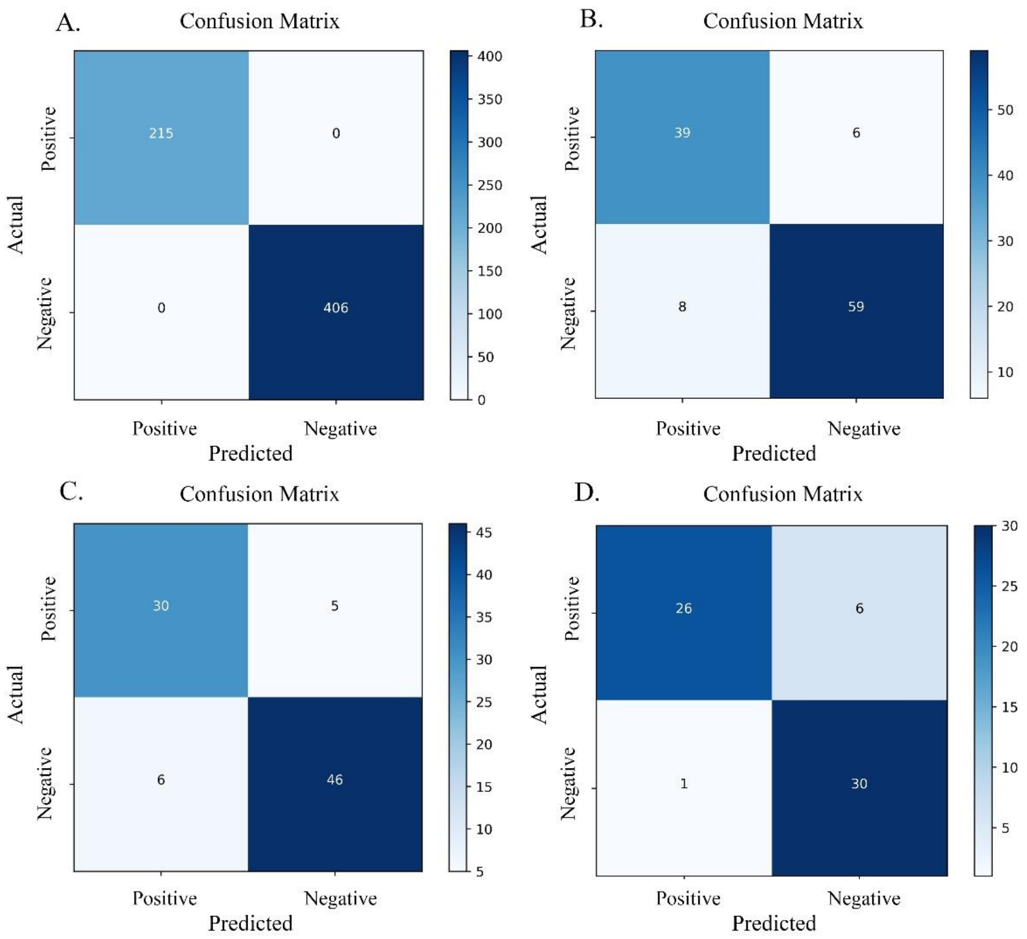

Supplementary Figure 4 Architecture of ResNet50. Conv, convolutional; ALN, axillary lymph node

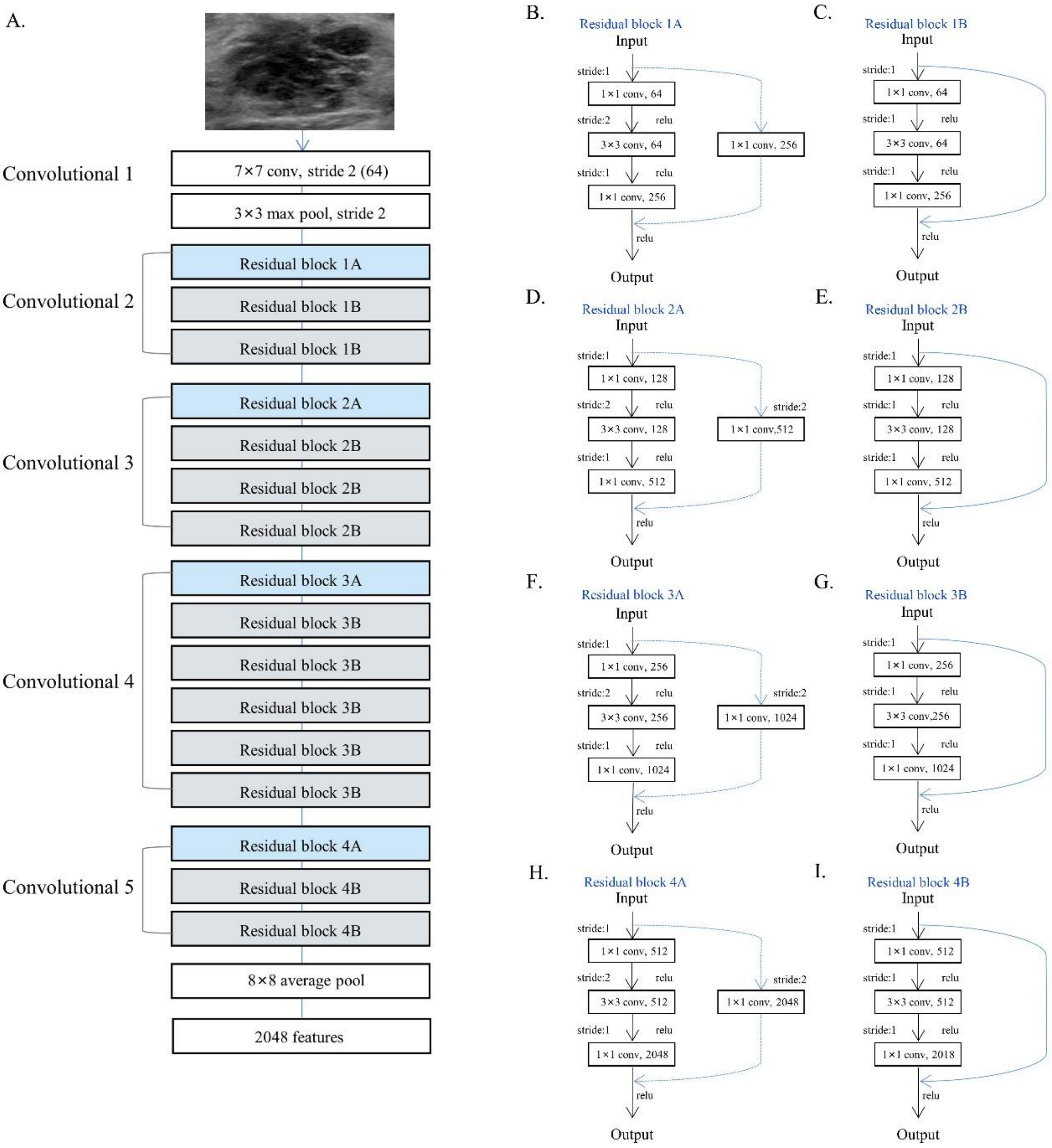

Supplementary Figure 5 Workflow of patient recruitment (A) and reproducibility evaluation (B). BI-RADS, Breast Imaging and Reporting Data Systems; ROI, region of interest.

A.

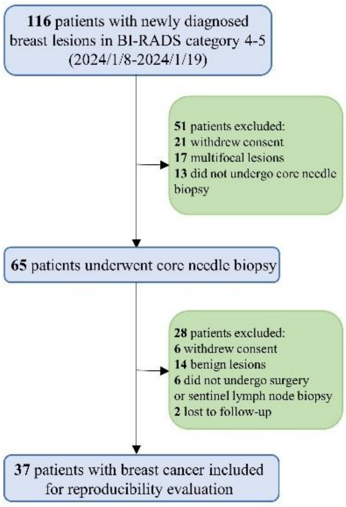

B.

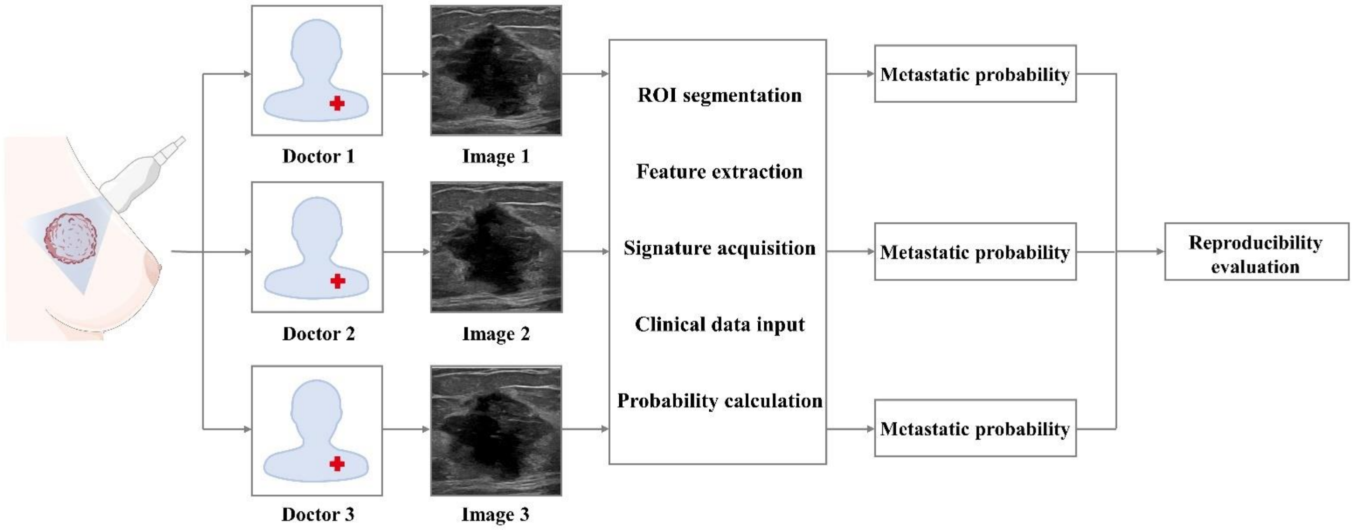

Supplement: Supplementary file 1 — Supplementary material [file 41523_2024_628_MOESM1_ESM.pdf]
