# Supplementary material for: Deep learning radiomics based prediction of axillary lymph node metastasis in breast cancer
Source: NPJ Breast Cancer. 2024 Mar 12;10:22. doi: 10.1038/s41523-024-00628-4 (PMC10933422; doi:10.1038/s41523-024-00628-4)
Supplement: Supplementary file 2 — Reporting summary [file 41523_2024_628_MOESM2_ESM.pdf]

Corresponding author(s): Wentao Kong and Luying Gui

Last updated by author(s): 2024/2/23

Reporting Summary

Nature Portfolio wishes to improve the reproducibility of the work that we publish. This form provides structure for consistency and transparency in reporting. For further information on Nature Portfolio policies, see our Editorial Policies and the Editorial Policy Checklist.

Statistics

For all statistical analyses, confirm that the following items are present in the figure legend, table legend, main text, or Methods section.

n/a	Confirmed	
☐	☒	The exact sample size (*n*) for each experimental group/condition, given as a discrete number and unit of measurement
☐	☒	A statement on whether measurements were taken from distinct samples or whether the same sample was measured repeatedly
☐	☒	The statistical test(s) used AND whether they are one- or two-sided<br>*Only common tests should be described solely by name; describe more complex techniques in the Methods section.*
☐	☒	A description of all covariates tested
☐	☒	A description of any assumptions or corrections, such as tests of normality and adjustment for multiple comparisons
☐	☒	A full description of the statistical parameters including central tendency (e.g. means) or other basic estimates (e.g. regression coefficient) AND variation (e.g. standard deviation) or associated estimates of uncertainty (e.g. confidence intervals)
☐	☒	For null hypothesis testing, the test statistic (e.g. *F*, *t*, *r*) with confidence intervals, effect sizes, degrees of freedom and *P* value noted<br>*Give P values as exact values whenever suitable.*
☒	☐	For Bayesian analysis, information on the choice of priors and Markov chain Monte Carlo settings
☐	☒	For hierarchical and complex designs, identification of the appropriate level for tests and full reporting of outcomes
☒	☐	Estimates of effect sizes (e.g. Cohen's *d*, Pearson's *r*), indicating how they were calculated

Our web collection on statistics for biologists contains articles on many of the points above.

Software and code

Policy information about availability of computer code

Data collection	No software was used.
Data analysis	All statistical analyses were conducted in R 4.1.2, Python 3.6, and MATLAB R2021b.

For manuscripts utilizing custom algorithms or software that are central to the research but not yet described in published literature, software must be made available to editors and reviewers. We strongly encourage code deposition in a community repository (e.g. GitHub). See the Nature Portfolio guidelines for submitting code & software for further information.

Data

Policy information about availability of data

All manuscripts must include a data availability statement. This statement should provide the following information, where applicable:

- Accession codes, unique identifiers, or web links for publicly available datasets
- A description of any restrictions on data availability
- For clinical datasets or third party data, please ensure that the statement adheres to our policy

The readers can send emails to the corresponding author to access the original images and clinical data for a reasonable course.

Research involving human participants, their data, or biological material

Policy information about studies with human participants or human data. See also policy information about sex, gender (identity/presentation), and sexual orientation and race, ethnicity and racism.

Reporting on sex and gender	The study was conducted only on women because it was a retrospective research to predict axillary lymph node metastasis for breast cancer.
Reporting on race, ethnicity, or other socially relevant groupings	Participants included in this study were all Asians because the research was conducted in China.
Population characteristics	Women with histologically diagnosed unifocal breast cancer were included in this study. They all underwent breast surgery and axillary lymph node dissection/ sentinel lymph node biopsy.
Recruitment	Patients were retrospectively enrolled. The inclusion criteria were listed as follows: (a) women with histologically diagnosed unifocal breast cancer, (b) patients with confirmed ALN status by ALN dissection/SLN biopsy, and (c) patients received ultrasound examination within 1 week before surgery.
Ethics oversight	Nanjing Drum Tower Hospital

Note that full information on the approval of the study protocol must also be provided in the manuscript.

Field-specific reporting

Please select the one below that is the best fit for your research. If you are not sure, read the appropriate sections before making your selection.

☒ Life sciences ☐ Behavioural & social sciences ☐ Ecological, evolutionary & environmental sciences

For a reference copy of the document with all sections, see nature.com/documents/nr-reporting-summary-flat.pdf

Life sciences study design

All studies must disclose on these points even when the disclosure is negative.

Sample size	Patients with histologically confirmed primary breast cancer in four hospitals from 2016/4 to 2022/6 were included in this study.
Data exclusions	The exclusion criteria were as follows: (a) patients who received neoadjuvant radiotherapy, chemotherapy, or other therapies preoperatively, (b) patients with ultrasound-invisible or non-mass-type lesions, (c) patients with multifocal lesions or insufficient image quality, (d) patients with metastatic breast cancer, and (e) patients with incomplete clinical or histopathological information. Multifocal lesions were excluded due to the difficulty of distinguishing the responsible lesion which caused metastasis from various masses.
Replication	Reproducibility of the model we constructed was assessed in 37 patients who were prospectively enrolled from Hospital I between January 8, 2024, and January 19, 2024. The inter-observer class correlation coefficient was calculated to assess model reproducibility. The result turned out that the model was of good reproducibility.
Randomization	It is not relevant to this study because all patients included underwent the same treatment.
Blinding	The investigators analyzed data in a blind manner.

Reporting for specific materials, systems and methods

We require information from authors about some types of materials, experimental systems and methods used in many studies. Here, indicate whether each material, system or method listed is relevant to your study. If you are not sure if a list item applies to your research, read the appropriate section before selecting a response.

Materials & experimental systems

n/a	Involved in the study	
☒	☐	Antibodies
☒	☐	Eukaryotic cell lines
☒	☐	Palaeontology and archaeology
☒	☐	Animals and other organisms
☐	☒	Clinical data
☒	☐	Dual use research of concern
☒	☐	Plants

Methods

n/a	Involved in the study	
☒	☐	ChIP-seq
☒	☐	Flow cytometry
☒	☐	MRI-based neuroimaging

Clinical data

Policy information about clinical studies

All manuscripts should comply with the ICMJE guidelines for publication of clinical research and a completed CONSORT checklist must be included with all submissions.

Clinical trial registration	Not available, it's a retrospective observational study.
Study protocol	Not available, it's a retrospective observational study.
Data collection	A total of 883 eligible patients with breast cancer who underwent preoperative breast and axillary ultrasound were retrospectively enrolled between April 1, 2016, and June 30, 2022.
Outcomes	Axillary lymph node metastasis status

Plants

Seed stocks	Not available
Novel plant genotypes	Not available
Authentication	Not available
